# The zinc-finger protein OEF-1 stabilizes histone modification patterns and promotes efficient splicing in the *Caenorhabditis elegans* germline

**DOI:** 10.1093/g3journal/jkab329

**Published:** 2021-09-14

**Authors:** Catherine E McManus, Mariateresa Mazzetto, Guifeng Wei, Mei Han, Valerie Reinke

**Affiliations:** 1 Department of Genetics, Yale University School of Medicine, New Haven, CT 06520, USA; 2 Developmental Epigenetics, Department of Biochemistry, University of Oxford, Oxford OX1 3QU, UK

**Keywords:** germ cells, chromatin, splicing, X chromosome, *C. elegans*

## Abstract

To ensure stable transmission of genetic information to the next generation, germ cells frequently silence sex chromosomes, as well as autosomal loci that promote inappropriate differentiation programs. In Caenorhabditis *elegans*, silenced and active genomic domains are established in germ cells by the histone modification complexes MES-2/3/6 and MES-4, which promote silent and active chromatin states, respectively. These states are generally mutually exclusive and modulation of one state influences the pattern of the other. Here, we identify the zinc-finger protein OEF-1 as a novel modifier of this epigenetic balance in the *C. elegans* germline. Loss of *oef-1* genetically enhances *mes* mutant phenotypes. Moreover, OEF-1 binding correlates with the active modification H3K36me3 and sustains H3K36me3 levels in the absence of MES-4 activity. OEF-1 also promotes efficient mRNA splicing activity, a process that is influenced by H3K36me3 levels. Finally, OEF-1 limits deposition of the silencing modification H3K27me3 on the X chromosome and at repressed autosomal loci. We propose that OEF-1 might act as an intermediary to mediate the downstream effects of H3K36me3 that promote transcript integrity, and indirectly affect gene silencing as a consequence.

## Introduction

Germ cells require a precise balance between gene expression and repression that largely depends upon the establishment of distinct chromatin states across the genome. Two important histone modifications contribute to the formation of these states: H3K27me3 marks repressive domains, where it limits chromatin accessibility to transcriptional regulators (van Mierlo *et al.* 2019), while H3K36me3 is present at transcribed genes, where it suppresses cryptic transcription initiation and promotes mRNA splicing, RNA processing, and DNA repair (Meers *et al.* 2017; Li *et al.* 2019). In *Caenorhabditis elegans*, these modifications are established via the chromatin-modifying pathway consisting of the histone methyltransferase (HMT) MES-4 and the Polycomb Repressive Complex 2 (PRC2) orthologs MES-2/3/6 (Bender *et al.*^2004^, ^2006^; Gaydos *et al.* 2012). MES-4 promotes H3K36me3 accumulation on autosomes, which leads to concentration of H3K27me3 on the X by MES-2/3/6 (Gaydos *et al.* 2012). Loss of MES activity results in inappropriate activation of X-linked genes and second-generation sterility (Capowski *et al.* 1991; Bender *et al.* 2006). The two marks occupy mutually exclusive domains of the genome (Gaydos *et al.* 2012; Evans *et al.* 2016), leading to a model in which the presence of one modification prevents the accumulation of the other at specific loci. However, beyond the core methyltransferase complexes, few factors that might influence this relationship between H3K27me3 and H3K36me3 have been identified.

We previously characterized the zinc-finger protein OEF-1 (Oocyte-Excluded Factor 1) as a novel, germline-specific nuclear factor that is required for a normal rate of progression through germ cell development (McManus and Reinke 2018). Germ cells lacking OEF-1 activity display precocious proliferation, an activated synaptic checkpoint, and more rapid progression through differentiation (McManus and Reinke 2018). However, the molecular activity of OEF-1 that might underlie these phenotypes is unknown. We previously demonstrated that OEF-1 is nuclear and preferentially associates with autosomal genes expressed in the germline in a distinctive distributed pattern across gene bodies. Indeed, the low, broad profile of OEF-1 binding can be seen on most germline-expressed genes, even for those that do not reach statistical significance in peak-calling methods. This observation suggests that OEF-1 might directly affect transcript levels of the genes at which it is located. Alternatively, OEF-1 might have a chromatin-associated function that indirectly affects downstream germline processes through another mechanism. Here, we set out to distinguish between these possibilities.

We first determine that OEF-1 has minimal effect on transcript abundance in germ cells, with mild reduction of X-linked transcript abundance. Consistent with this observation, loss of OEF-1 increases the silencing mark H3K27me3 throughout the genome, particularly on the X chromosome. However, OEF-1 binding primarily correlates with the presence of the activating histone modification H3K36me3, and OEF-1 is necessary to prevent H3K36me3 levels from decreasing in *mes-4* mutants. Moreover, loss of OEF-1 activity results in reduced splicing efficiency of germline-expressed mRNAs. These observations together suggest that OEF-1 might assess or interpret H3K36me3 levels to mediate downstream functions such as splicing that are associated with productive gene expression. We hypothesize that this activity would indirectly affect gene repression by disrupting the balance between activating and silencing modifications.

## Materials and methods

### *Caenorhabditis elegans* strains

Strains used in this study are listed in Supplementary Table S7. Strains were maintained by standard methods on NGM plates seeded with OP50 bacteria as described (Brenner 1974). Bristol N2 was used as the wild-type reference strain. All growth was performed at 20°C, except for *glp-4* strains which were maintained at 15°C and shifted to 25°C before collection. For balanced strains, larval stage 4 (L4) worms expressing pharynx GFP were picked to ensure the selection of first-generation homozygotes in downstream assays.

### Brood size analyses

L4 worms were singly placed on seeded plates and moved to fresh plates each day until embryo production ceased. Unhatched embryos were scored as dead 24 h after the mother was removed. Live larvae were counted two days later. To score the presence or absence of germlines, L4s were aged 8–9 h before fixation in 20 μl of Carnoy's fix (300 μl 100% ethanol, 150 μl chloroform, and 50 μl acetic acid) on Super Frost slides. Twelve microliters of 1 ng/mL DAPI diluted in M9 was added to coverslips, which were touched to slides and sealed with nail polish before scoring.

### Immunostaining

Adults 16–20 h post-L4 were dissected to release gonads in 1.1x egg salts (10X egg salts: 250 mM HEPES pH 7.4, 1.18 M NaCl, 20 mM MgCl2, 20 mM CaCl2, 4.8 mM KCl) with 0.1% Tween 20 and 0.3 mM levamisole. Gonads were fixed with 1% formaldehyde, then freeze-cracked on a metal block embedded in dry ice and immediately placed in −20°C methanol. Blocking was performed using 0.5% BSA in PBST for 30 min at room temperature. For histone modification staining, the area around the gonads was dried with a Kim wipe and a circle was drawn around the tissue using a mini PAP pen before the addition of the primary antibody. Primary and secondary antibody incubations were performed overnight at 4°C in a 50 μl volume. The following antibody dilutions were used: 1:50 anti-OEF-1 (McManus and Reinke 2018); 1:2000 anti-GFP (Abcam ab13970); 1:20 anti-H3K4me2 (EMD Millipore CMA303); 1:200 anti-H3K27me3 (EMD Millipore 07-449); 1:1000 anti-H3K36me3 (Abcam ab9050). Secondary antibodies (Molecular Probes) were diluted 1:500. Slides were mounted in Vectashield anti-fade mounting medium (Vector Laboratories) with no. 2 coverslips (Corning) and sealed with nail polish. 0.375 μm Z stacks were acquired using a Zeiss Axioplan microscope with a 20X, 40X, or 100X objective, and a Zeiss AxioCam MRm camera, and processed using Axiovision software.

For H3K27me3 mean intensity measurements, ImageJ (NIH) was used to make Z projection images. The X chromosome was identified in germ cells by lack of H3K4me2 staining and enrichment for H3K27me3 staining. In each germ cell in which a clear X chromosome could be identified, a representative autosome was identified by high levels of H3K4me2 and low levels of H3K27me3. The free draw tool was used to outline a representative X and a representative autosome in wild-type and *oef-1(vr25)* germ cells. The mean intensity of H3K27me3 in the outlined areas was recorded. The measurements were repeated for 6–8 nuclei per gonad, 7 gonads per genotype (46–48 nuclei total).

For the H3K36me3 proliferative zone reduction phenotype, extruded and well-stained gonads were scored as having no reduction, slight reduction, or strong reduction in H3K36me3 staining in the proliferative zone as compared to pachytene in the same gonad. The genotypes of the slides were blinded before scoring. The slides represented two or three independent staining experiments all scored in the same sitting.

### Germ nuclei isolation

Germ nuclei isolation was performed as in Han *et al.* (2019). N2 and *oef-1(vr25)* worms were grown to gravid on NGM plates, bleached, and hatched overnight in M9 for at least 24 h. Fifty thousand L1s were plated on enriched plates and grown until most worms had ≥4 embryos. Around 1 million worms were used per nuclei collection. The worms were washed off with M9 in sets of 6 plates into 50 mL falcon tubes, washed twice with M9, then were fixed for 28 min in 2% formaldehyde diluted in M9. After a wash with 1 M Tris (pH 7.5), the worms were washed twice more with M9. The worms were then washed with chilled Nuclei Purification Buffer (NPB) (50 mM HEPES pH 7.5, 40 mM NaCl, 90 mM KCl, 2 mM EDTA, 0.5 mM EGTA, 0.1% Tween 20, 0.2 mM DTT, 0.5 mM PMSF, 0.5 mM spermidine, 0.25 mM spermine, and proteinase inhibitor cocktail—1 tablet per 25 mL NPB).

The fixed and washed worms were resuspended in NPB and transferred to prechilled 7 mL glass Dounce homogenizers (Wheaton). One Dounce homogenizer was used per set of 6 plates. Fifteen loose strokes were followed by 30 tight strokes with a quarter turn between each Dounce. The worms were transferred into chilled 50 mL falcon tubes, and NPB was added to 30 mL. The falcon tubes were vortexed on medium-high speed for 30 s, followed by 15 min on ice. The vortex and ice incubation were repeated. The worms were then passed through three 40 μm filters (Fisherbrand) followed by eight 20 μm filters (pluriSelect). The nuclei were spun at 3100 rpm for 6 min at 4°C. The supernatant was removed, and the nuclei were resuspended in 1 mL NPB and transferred to a nonstick 1.5 mL tube (Ambion). A 5 μl sample of nuclei was removed, incubated with DAPI, and counted using a hemacytometer (Hausser Scientific). Finally, the nuclei were spun at 4°C at 4000 rpm for 5 min, the supernatant was removed, and the nuclei were flash frozen in liquid nitrogen. The nuclei were stored at −80°C until sonication. 2–3 nuclei collections were pooled per ChIP experiment (for a total of 15–20 million nuclei per IP).

### ChIP-sequencing

Isolated germ cell nuclei were thawed on ice, and 120 μl of Nuclear Lysis Buffer (50 mM Tris pH 8.0, 10 mM EDTA, 1% SDS, 0.5 mM PMSF, proteinase inhibitor cocktail) was added to each sample. The nuclei were vortexed vigorously for 1 min followed by 1 min on ice. The vortex step was repeated. The nuclei were sonicated at 2°C in a water bath sonicator (Misonix S-4000) at 20% amplitude, with 10 s on/10 s off pulses for a total process time of 20 min.

1.2 mL of FA buffer (50 mM HEPES pH 7.5, 1 mM EDTA, 1% Triton X-100, 0.1% sodium deoxycholate, 150 mM NaCl, 1 mM DTT, 0.5 mM PMSF, proteinase inhibitor cocktail) was added to each sonicated sample. A 1:20 volume of 20% sarkosyl was added, and the samples were spun at 13,000 × g for 5 min at 4°C. The supernatant was transferred to a new tube, and then 5% of the lysate was removed for the input sample. The input samples were stored at −20°C overnight. Five micrograms of anti-H3K27me3 (Active Motif MABI0323) or anti-H3K36me3 (Active Motif MABI0333) was incubated with each IP sample overnight at 4°C with rotation.

The next day, the input samples were thawed on ice and were incubated with 2 μl of 10 mg/mL RNase A for 2 h at room temperature. Approximately 40 μl of Protein G Sepharose beads (GE Healthcare) were washed 4 times with 1 mL FA, spinning for 2 min at 2500 × g between each wash. The entire IP sample was added directly to the beads, and the samples were rotated for 2 h at 4°C.

After the RNase A treatment of the input samples, 230 μl of elution buffer (1% SDS in TE, 250 mM NaCl) was added to each input sample to bring the total volume up to 300 μl. 2.05 μl of 19.5 mg/mL proteinase K was added, and the input samples were incubated at 55°C for 3 h.

After the 2-h bead incubation, the beads were washed at room temperature as follows (1 mL for each wash): 2 times with FA for 5 min each, 1 time with FA with 500 mM NaCl for 10 min, 1 time with TEL (0.25 M LiCl, 1% NP40, 1% sodium deoxycholate, 1 mM EDTA, 10 mM Tris pH 8.0) for 10 min, 2 times with TE for 5 min each. One hundred and fifty microliter of elution buffer was added to each sample and was incubated at 65°C for 15 min with occasional vortexing. The elution step was repeated and the eluates were pooled. 1.03 μl of 19.5 mg/mL proteinase K was added to the eluates and the incubation was performed at 55°C for 1 h. After the proteinase K digestion, input and IP samples were incubated overnight at 65°C to reverse the crosslinks. The next day, input and ChIP DNA were purified using the QIAquick PCR Purification Kit. Samples were eluted in 40 μl TE and submitted for library preparation and sequencing at the Yale Center for Genome Analysis.

Somatic samples were prepared as described (Kudron *et al.* 2018). *glp-4* and *glp-4; oef-1* animals were grown at the permissive temperature of 15°C and embryos were isolated by bleaching and hatching overnight in M9 at 15°C. Forty thousand L1s were plated on peptone-enriched plates and were grown at 25°C until the young adult stage. Adult worms were washed off plates with M9 and were washed 3 times, spinning at 3100 × g between each wash. Worms were fixed in 50 mL 2% formaldehyde in M9 for 28 min at room temperature. After spinning, worms were washed with 50 mL 1 M Tris, pH 7.5 followed by two more washes with M9. A final wash with FA buffer was performed before pellets were flash-frozen and stored at −80°C. After thawing on ice, pellets were resuspended in 750 μl FA buffer and transferred to a 2 mL Kontes dounce (Kimble Chase). Samples were dounced 15 times for two cycles with the A pestle and 15 times with the B pestle for four cycles with a 1 min hold between each cycle. Pellets were sonicated using a SFX250 sonifier (Branson) in 1.5 mL FA buffer in an ice bath at 22% amplitude with 10 s on/1 min off pulses for a total process time of 5 min 40 s. Sonicated samples were transferred to nonstick tubes and spun at 13,000 × g for 15 min at 4°C. 4.4 mg of lysate was used per IP in a volume of 400 μl. 1:20 volume of 20% Sarkosyl solution was added to each 400 μl sample and the samples were spun at 13,000 × g for 5 min at 4°C. The supernatant was transferred to new nonstick tubes. The rest of the ChIP protocol was performed as for isolated germ nuclei.

### Sequencing and data analysis

Seventy-five-bp single-end reads were sequenced on an Illumina HiSeq2500 in rapid-run mode. The raw ChIP and corresponding input fastq sequencing reads were mapped to the genome (version ce10) by Bowtie2 (v2.3.2) (Langmead and Salzberg 2012) with default parameters. The datasets for two replicates were merged for further analysis. To eliminate the replicate bias, the alignment file (bam) from the sample with larger library size was downsampled to the size of the replicate with smaller library size, and then merged together by Samtools (v1.3) (Li *et al.* 2009). Peaks were called by MACS2 (v2.1.1) (Zhang *et al.* 2008) with the key parameter (-q 0.001 –nomodel –extsize 200) against the merged input. Normalization was performed by bamCompare, which scales the IP to input using read number, and wig files were generated using a custom script (https://github.com/guifengwei/glib/blob/master/bam2wig.py), which also scales IP to input using read number. Heatmaps were generated by ngs.plot.r (v2.63) (Shen *et al.* 2014) with the default parameters except (-G ce10 -R genebody -SC global). Genes longer than 1 kb were kept for analysis. The germline-enriched genes were defined as in (Han *et al.* 2019). Metagene plots were generated by computing matrices binned into 50 bp windows for histone modifications or 100 bp windows for OEF-1 using computeMatrix v2.5.0 and plotProfile v2.5.0 in deepTools (Ramírez *et al.* 2016). The correlation plot was generated using multiBigwigSummary followed by plotCorrelation in deepTools. The genomic regions used were all genes including 2 kb up/downstream of the TSS/TES. Supplementary Table S8 lists all samples used for genomic analyses.

### RNA-sequencing

N2 and *oef-1(vr25)* worms were grown to gravid on large NGM plates, bleached, and hatched overnight in M9 in the absence of food. The next day, 200 L1s were plated on small NGM plates and were grown to young adulthood. One hundred young adults of each genotype were dissected to release gonads in M9 with 0.1% levamisole and 0.1% Tween 20, cut at the spermatheca, and transferred in as little liquid as possible to 100 μl of TRIzol (Invitrogen) on ice. The samples in TRIzol were flash frozen in liquid nitrogen and were stored at −80°C until subsequent purification steps. Growths and dissections were repeated for a total of 400 gonads per replicate for each genotype.

Samples were thawed in a 37°C water bath, vortexed, and flash frozen again in liquid nitrogen. The freeze-thaw step was repeated twice more. Hundred microliters of chloroform was added, the samples were shaken for 15 s, and then left to sit for 8 min at room temperature. Samples were spun at 12,000 × g for 20 min at 4°C. The top aqueous layer was removed to a fresh tube, then an equal volume of isopropanol was added and incubated at −20°C overnight. The next day, the samples were spun at 12,000 × g for 30 min at 4°C. The supernatant was discarded, and the pellets were washed with 500 µl of 75% ethanol and spun at full speed at 4°C for 5 min. The wash and spin steps were repeated. As much ethanol was removed as possible, and the pellets were dried for 5 min. Finally, the pellets were resuspended with 20 µl of RNase-free water preheated to 55°C. DNase treatment was performed using the Ambion DNA-free kit according to the manufacturer’s instructions. DNase-treated samples were then cleaned up using the RNeasy Mini Kit and eluted in 50 µl of RNase-free water.

Ribozero library preparation was performed at the Yale Center for Genome Analysis. Seventy-five bp paired-end reads were sequenced on an Illumina HiSeq2500. Trimmed reads were mapped to WS220 using TopHat v2.0.14 (Trapnell *et al.* 2012; Kim *et al.* 2013) and assembled using Cufflinks v2.2.1 and Cuffmerge v1.1.4 (Trapnell *et al.* 2010). Differential expression testing between N2 and *oef-1(vr25)* assemblies was performed using CuffDiff v2.2.1 (Trapnell *et al.* 2013) and DESeq2 (Love *et al.* 2014).

For the intron retention analysis, RNA-seq paired-end reads were aligned to ce10 reference genome using HISAT2 (Kim *et al.* 2019) with default parameters. Reads were then assembled using two independent methods: IntEREst package (Oghabian *et al.* 2018) and Rsubread (Liao *et al.* 2019). Both were used to count intron and splice junction reads, using minOverlap of 60 bp for the latter. Intron retention differential analysis was performed using DESeq2 (Anders and Huber 2010) with default parameters and a multi-factor design (condition + exon-intron): the samples from the “control” dataset were mixed and a correction for batch effect was applied. Visualization of results and generally applicable gene enrichment (GAGE) analysis were performed using ggplot2 (Wickham 2016) and gage (Luo *et al.* 2009) packages on Bioconductor. RNA degradation was measured by a calculation of “transcript integrity”, which determines the frequency of defined ends of transcripts compared to shortened or incomplete transcripts, using RseQC software for Linux (Wang *et al.* 2016). Supplementary Table S8 lists all samples used for genomic analyses.

## Results

### Loss of OEF-1 activity has minor effects on germline gene expression

**Figure 1:**
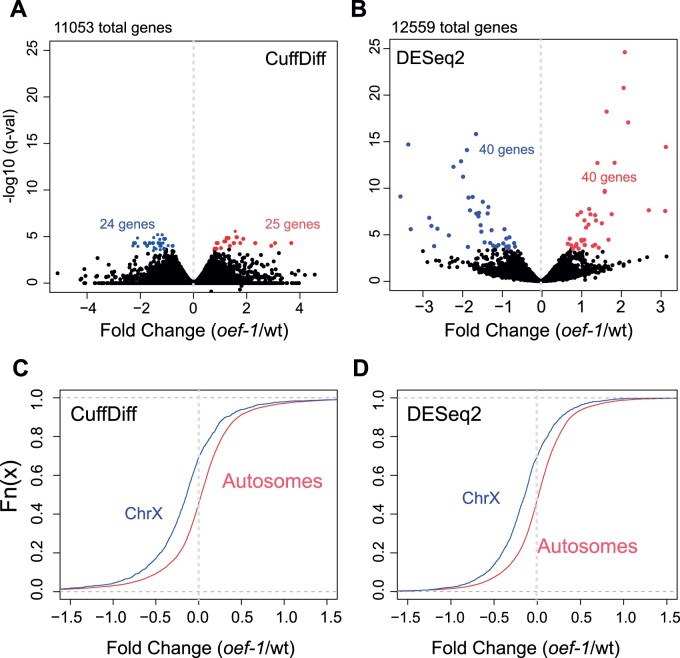
*oef-1* mutants display few changes in transcript abundance but a global decrease in X-linked expression. (A,B) Volcano plots showing fold change of gene expression in *oef-1(vr25)* mutants (*x*-axis) compared to wild type relative to log10 of *q*-value (*y*-axis) using either CuffDiff (A) or DESeq2 (B) programs. Blue dots show significantly downregulated genes, and red indicates significantly upregulated; cases with *q*-value <0.05 (CuffDiff) and *p*adj <0.05 (DESeq2) were counted as significant. Data are from the combined analysis of three biological replicates. (C, D) Cumulative plot showing the distribution of gene expression changes for X-linked (blue) and autosomal (red) transcripts in *oef-1(vr25)* dissected gonads relative to wild-type from the CuffDiff analysis (C, *N* = 1921 X-linked genes, 12,270 autosomal genes) and DESeq2 analysis (D, *N* = 1565 X-linked genes, 10,994 autosomal genes). Only genes with a log2 change of less than 1.5-fold or more than −1.5-fold are shown. Data are from the combined analysis of three biological replicates. *P* < 2.2^e−16^, Wilcoxon test.

In order to gain more insight into the function of OEF-1 in the germline, we first determined whether loss of *oef-1* results in significant changes in transcript abundance in gonads. We used the previously defined *oef-1(vr25)* allele, which contains a 56-bp frameshift deletion in exon 2 generated by CRISPR/Cas9 gene editing (McManus and Reinke 2018). We initially used CuffDiff to perform differential expression analysis of three replicates of RNA-sequencing of dissected adult gonads from wild-type and *oef-1(vr25)* mutants and found relatively few significant changes in gene expression (Figure 1A). We therefore also applied DESeq2 to determine whether this result was consistent regardless of computational approach. Again, we saw few transcripts that were reproducibly and significantly up- or downregulated in all three replicates of RNA-seq (Figure 1B). Comparison between the two analyses identified only 37 genes with significantly different expression identified by both programs, and these genes exhibited no obvious similarities in chromosomal location, gene function, or expression patterns (Supplementary File S1). Moreover, of the 37 genes, only 12 are directly bound by OEF-1 (Supplementary File S1). Together, these analyses indicate that OEF-1 does not have a consistent, significant effect on transcript abundance of the vast majority of its autosomal target genes. However, although few individual X-linked genes exhibited statistically significant changes in gene expression, we found a subtle yet global decrease in X chromosome gene expression in *oef-1(vr25)* compared to wild type that was not detected for autosomes using either the CuffDiff or DESeq2 datasets (Figure 1, C and D). This result suggests that X-linked transcripts, which are already present at very low levels in germ cells (Kelly *et al.* 2002), are further reduced upon loss of OEF-1.

### *oef-1* mutant germ nuclei exhibit increased enrichment of H3K27me3 on the X chromosome

**Figure 2:**
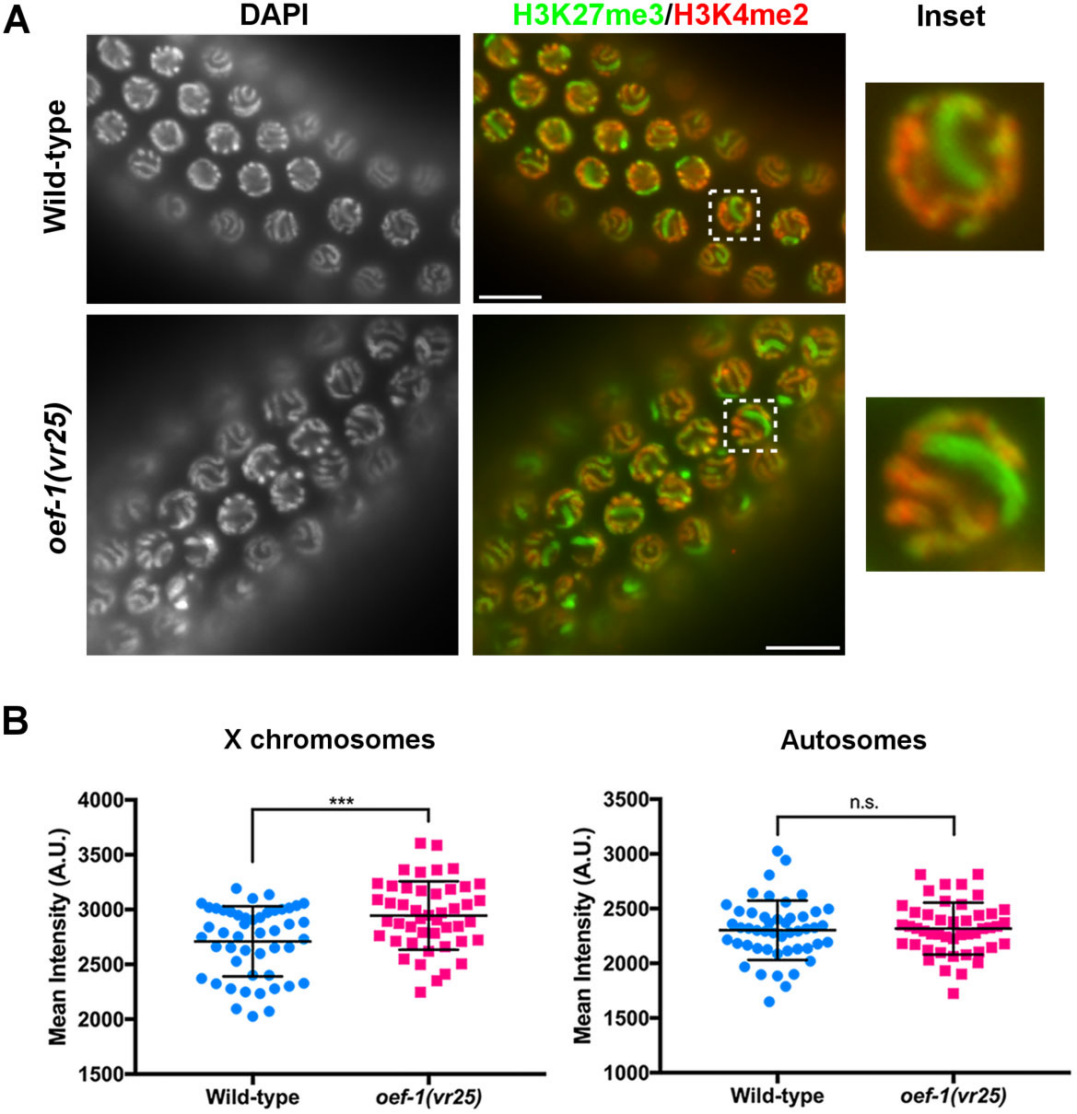
*oef-1* mutant germ cells exhibit higher enrichment of H3K27me3 on the X chromosome. (A) Representative wild-type and *oef-1(vr25)* pachytene nuclei co-stained with the X chromosome-enriched histone modification H3K27me3 (green) and the autosomal-specific histone modification H3K4me2 (Kelly *et al.* 2002) (red). DAPI (DNA) is at left. Box indicates inset at right. Scale bars, 10 μm. (B) Quantification of H3K27me3 staining. The mean intensities of H3K27me3 staining for X chromosomes (left) were measured in wild-type (2710 A.U.) and *oef-1(vr25)* (2946 A.U.) pachytene nuclei. H3K27me3 intensity was equivalent between wild-type (2302 A.U.) and *oef-1(vr25)* (2318 A.U.) for representative autosomes (right). ***P < 0.001, unpaired *t*-test. n.s., not significant. A.U., arbitrary units. *n* ≥ 6 nuclei from 7 gonads per genotype (≥ 46 nuclei total). Error bars represent S.D.

The effect on X chromosome gene expression was surprising because OEF-1 has little binding on the X (McManus and Reinke 2018). Given the established importance of H3K27me3 on X chromosome silencing (Gaydos *et al.* 2012), we wondered whether OEF-1 might affect H3K27me3 levels. We first performed immunostaining to monitor H3K27me3 in germ nuclei, and specifically observed detectable enrichment of H3K27me3 on the X chromosome relative to the autosomes as demonstrated previously (Gaydos *et al.* 2012). Strikingly, H3K27me3 staining of the X appeared even stronger in *oef-1(vr25)* mutant germ cells compared to wild type (Figure 2A). Quantification of the signal indeed detected a significant increase in the average mean H3K27me3 intensity for *oef-1(vr25)* X chromosomes compared to wild type (Figure 2B). On the other hand, there was no significant difference in the mean intensities of H3K27me3 staining between *oef-1(vr25)* and wild-type autosomes (Figure 2B). This result suggests that loss of OEF-1 leads to further enrichment of H3K27me3 on the X chromosome and is consistent with increased downregulation of X-linked gene expression in *oef-1* mutants.

### H3K27me3 accumulation increases genome-wide in *oef-1* mutant germ nuclei

In order to investigate the changes in H3K27me3 accumulation in *oef-1* mutants at the resolution of individual loci, we used a method to isolate germ nuclei (Han *et al.* 2019) from wild type and *oef-1(vr25)* adults, and then performed H3K27me3 chromatin immunoprecipitation followed by sequencing (ChIP-seq). As a soma-only control, we also performed H3K27me3 ChIP-seq on *glp-4(bn2)* and *glp-4; oef-1* whole animals, which lack germ cells at the restrictive temperature of 25°C (Beanan and Strome 1992). Because this isolation method was recently developed, we first confirmed that the wild-type H3K27me3 chromatin profiles accurately reflect expected germline and somatic patterns. Previous studies demonstrate that the X chromosome is enriched for H3K27me3 relative to autosomes in germ nuclei, and that any genes marked by H3K27me3, whether on the X or on autosomes, are likely to have very low expression levels in wild-type adult germ cells (Kelly *et al.* 2002; Reinke *et al.* 2004). We therefore first examined the H3K27me3 profile across all chromosomes and found that H3K27me3 is more broadly and evenly distributed across the X relative to autosomes in germ nuclei but not in the soma (Supplementary Figure S1A). In addition, the genes with enriched expression in the germline have relatively low H3K27me3 signal in isolated germ nuclei compared to genes with enriched expression in the soma (Supplementary Figure S1B). In particular, genes primarily expressed in meiotic or oogenic germ cells exhibit very low levels of H3K27me3 (Supplementary Figure S1C). Thus, these datasets accurately represent the known germline- and soma-specific patterns of H3K27me3.

**Figure 3:**
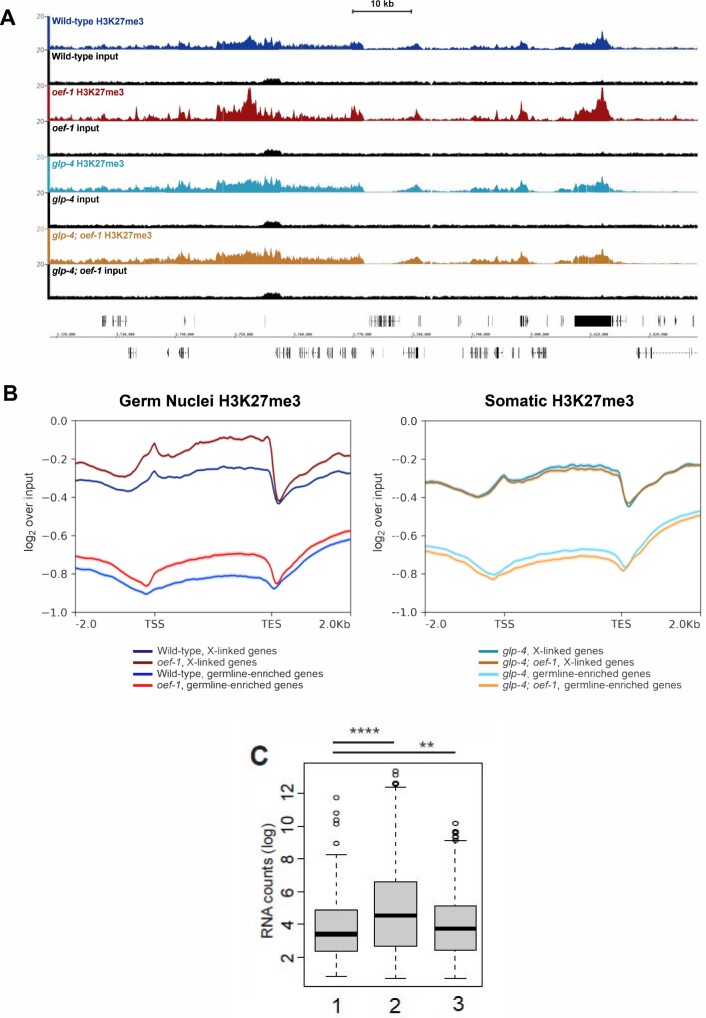
H3K27me3 accumulation increases genome-wide in *oef-1* mutant germ nuclei. (A) Browser view of H3K27me3 ChIP-seq on the X chromosome. Wild-type germ nuclei track is shown in navy, *oef-1* germ nuclei in maroon, *glp-4* soma in teal, *glp-4; oef-1* soma in dark orange. Input tracks in black are shown below each sample. Coordinates shown are chrX : 3,717,067–3,832,532. Scale bar, 10 kb. (B) Left: Metagene analysis of H3K27me3 ChIP-seq signal for wild-type (blue and navy) and *oef-1(vr25)* (red and maroon) isolated germ nuclei across either X-linked genes (maroon and navy) or germline-enriched autosomal genes (red and blue). ChIP-seq signal is log2 over input. Right: Metagene analysis of H3K27me3 ChIP-seq signal for *glp-4* (dark teal and light teal) and *glp-4; oef-1* (dark orange and light orange) somatic samples across either X-linked genes (dark teal, dark orange) or germline-enriched autosomal genes (light teal and light orange). TSS, transcription start site. TES, transcription end site. Shading indicates standard error. (C) Box plot showing significant difference in FPKM distribution for the 485 autosomal genes with increased H3K27me3 in *oef-1* mutants (“1”) compared to all autosomal genes (“2”) (****P < 2.2e-16) and to all X-linked genes (“3”) (**P = 0.004754), Wilcoxon test.

We next compared H3K27me3 accumulation patterns between wild type and *oef-1(vr25)* isolated germ nuclei and found elevated average levels of H3K27me3 on X-linked genes in *oef-1(vr25)* (Figure 3A, Supplementary Figure S2), which we confirmed by metagene and heatmap analysis (Figure 3B, Supplementary Figures S3–S6). Notably, the pattern of H3K27me3 accumulation in *oef-1* germ nuclei does not appear to spread or expand beyond wild-type regions (Figure 3A, Supplementary Figures S3–S6). The somatic datasets showed no change in average H3K27me3 levels on the X chromosome, which was expected given that OEF-1 expression is limited to germ cells (McManus and Reinke 2018) (Figure 3, A and B, Supplementary Figures S2–S6). These data are consistent with increased H3K27me3 accumulating on the X chromosome in germ but not somatic nuclei upon loss of OEF-1.

Notably, we also identified 485 autosomal genes with a greater than twofold increase in H3K27me3 levels in *oef-1(vr25)* mutant germ cells (Figure 3B, Supplementary Figure S5). This increase was likely not detectable by immunofluorescence (Figure 2B) because these genes are dispersed across the five autosomes. Of the 485 autosomal genes with higher H3K27me3 in *oef-1(vr25)* germ nuclei (Supplementary Table S1, Supplementary File S2), 82% are expressed either during spermatogenesis in the L4 stage or in the soma (Ortiz *et al.* 2014) and are therefore likely expressed at low levels in the adult germline. Indeed, their expression is significantly lower than the average of all autosomal genes and more similar to X-linked genes (Figure 3C). These data support an indirect role for OEF-1 in limiting the levels of H3K27me3 on the X chromosome and at autosomal loci enriched for that modification.

### Loss of OEF-1 has minimal effect on H3K36me3 levels in germ nuclei

Although OEF-1 affects H3K27me3 levels, OEF-1 is not localized to genomic regions harboring that modification but instead is present across gene bodies of most germline-expressed genes on autosomes (McManus and Reinke 2018), and therefore should be more closely associated with H3K36me3. Given the known balance between H3K27me3 and H3K36me3, we hypothesized that the change in H3K27me3 levels in *oef-1* mutants might occur as a response to changes in H3K36me3 levels. We therefore performed H3K36me3 ChIP-seq in isolated germ and somatic nuclei from wild type and *oef-1(vr25)* mutants. As before, we first confirmed that the wild-type H3K36me3 profile reflected the expected pattern for the germline and soma. Indeed, H3K36me3 levels were depleted from the X chromosome relative to autosomes in germ nuclei but not in the soma (Supplementary Figure S1A) (Gaydos *et al.* 2012). Moreover, H3K36me3 was enriched on genes known to be expressed in the germline at much greater levels than in the soma (Supplementary Figure S1, B and C). Finally, the H3K36me3 and H3K27me3 profiles are anti-correlated in isolated germ nuclei and in somatic tissues (−0.58 and −0.62, respectively, Pearson’s correlation coefficient; Supplementary Figure S1, D and E). Thus, the germline and somatic H3K36me3 datasets replicate the expected patterns in each tissue.

**Figure 4:**
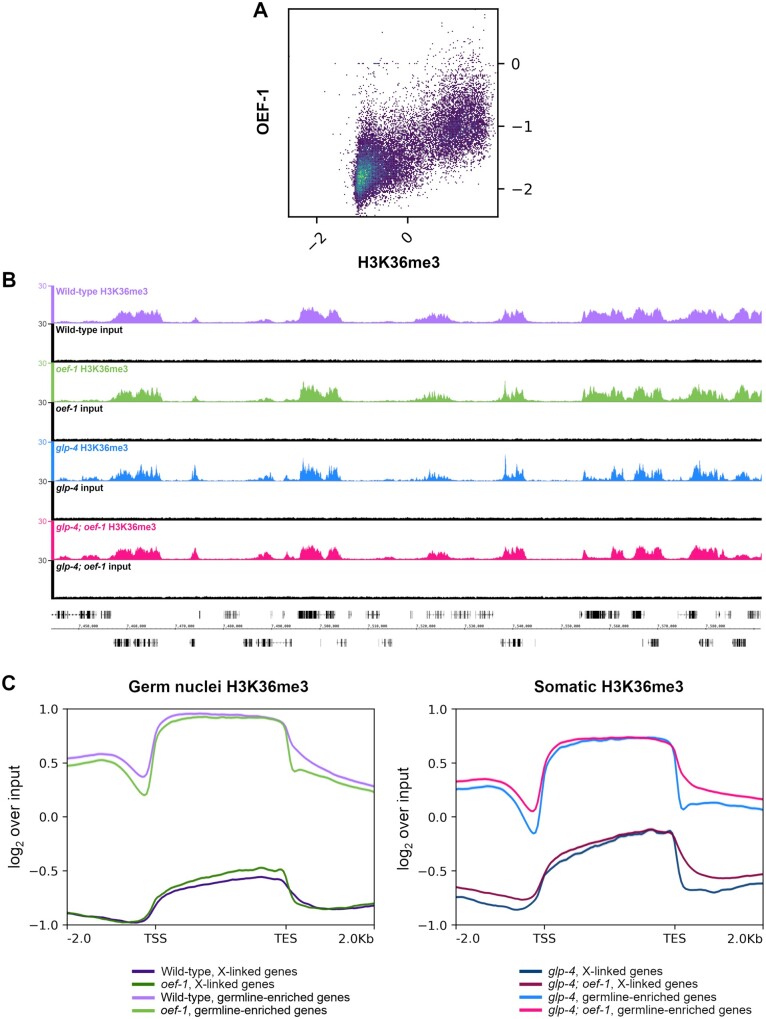
Loss of OEF-1 has minimal effect on H3K36me3 levels in germ nuclei. (A) Correlation plot comparing OEF-1 ChIP-seq signal (log2 over input) and wild-type germ nuclei H3K36me3 ChIP-seq (log2 over input) for all genes, including 2 kb up/downstream. Pearson’s correlation coefficient = 0.70. (B) Screenshot of H3K36me3 ChIP-seq signal on chromosome I. Wild-type germ nuclei track is shown in purple, *oef-1* germ nuclei in green, *glp-4* soma in blue, *glp-4; oef-1* soma in pink. Input tracks in black are shown below each sample. Coordinates shown are chrI : 7,441,886–7,591,919. Scale bar, 10 kb. (C) Left: Metagene analysis of H3K36me3 ChIP-seq signal for wild-type (purple) and *oef-1(vr25)* (green) isolated germ nuclei across either X-linked genes (dark purple, dark green) or germline-enriched autosomal genes (light purple and light green). ChIP-seq signal is log2 over input. Right: Metagene analysis of H3K36me3 ChIP-seq signal for *glp-4* (blue) and *glp-4; oef-1* (pink) somatic samples across either X-linked genes (navy, dark pink) or germline-enriched autosomal genes (blue and pink). TSS, transcription start site. TES, transcription end site. Shading indicates standard error.

We then examined the relationship between H3K36me3 levels and OEF-1 in wild-type germ nuclei. We found a strong positive genome-wide correlation between H3K36me3 and OEF-1 binding levels (0.7, Pearson’s correlation coefficient) (Figure 4A). The similarity in genomic profiles was also apparent across individual genes as viewed by genome browser (Supplementary Figure S7). We next determined whether loss of OEF-1 affected H3K36me3 levels. Visible differences in H3K36me3 abundance or pattern between wild type and *oef-1* mutants were not detected (Figure 4B). We therefore performed metagene analysis to try to identify more subtle differences (Figure 4C, Supplementary Figures S3–S6), which confirmed that OEF-1 does not lead to a significant alteration of H3K36me3 patterns or abundance across gene bodies for either germline-expressed or X-linked genes. At the 5’ and 3’ end of genes, there is a slight dip in H3K36me3 levels in *oef-1* germ nuclei relative to wild type. Strikingly, the inverse pattern is seen in somatic nuclei, which do not express OEF-1 (Figure 4C, Supplementary Figures S4–S6). Whether the change in H3K36me3 levels at gene ends has biological significance is currently unclear. Overall, loss of OEF-1 activity alone does not measurably affect the distribution or steady-state level of H3K36me3 in germ cells.

### OEF-1 acts redundantly with MES-4 to promote germline H3K36me3

**Figure 5:**
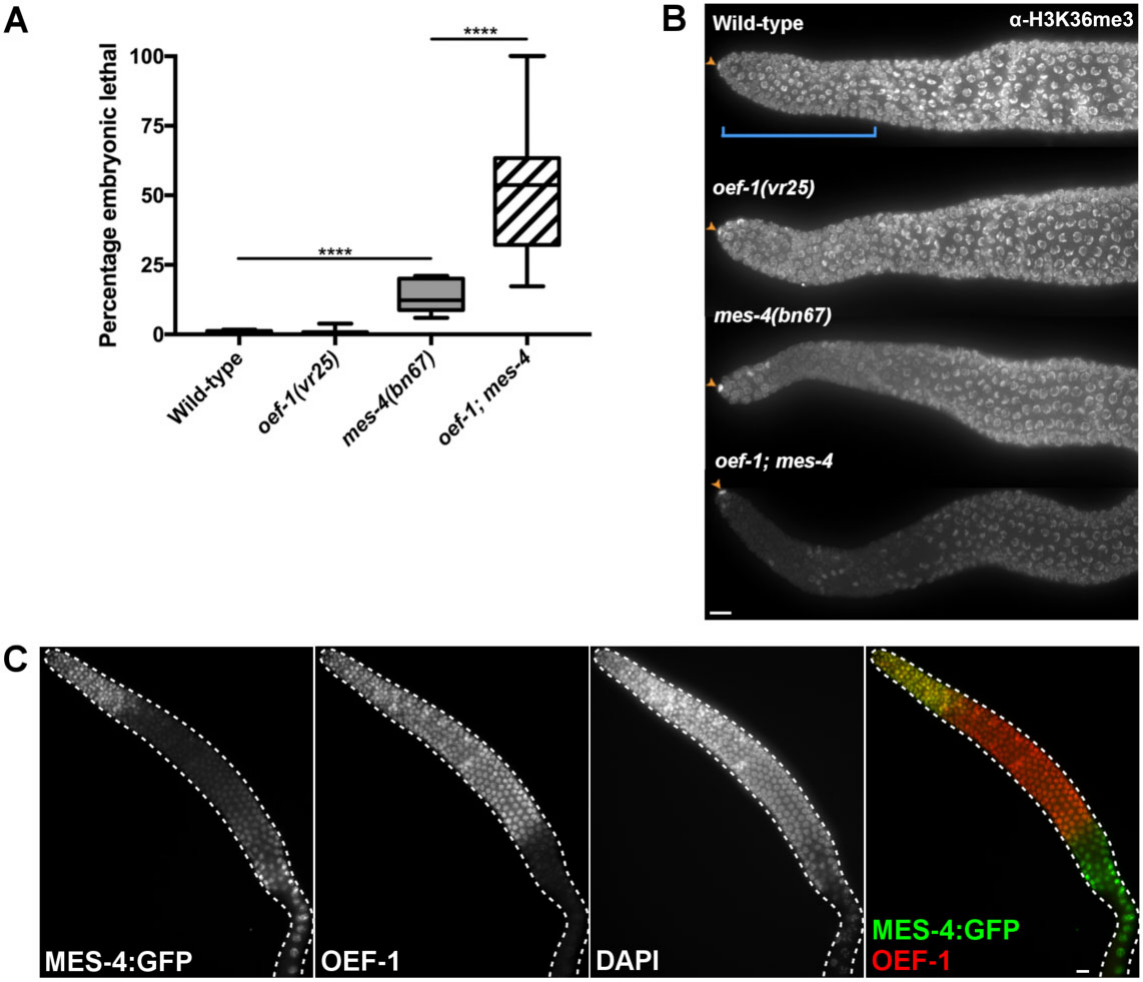
Loss of *oef-1* enhances *mes-4* phenotypes. (A) Quantification of embryonic lethality in *oef-1; mes-4* broods. *oef-1; mes-4* broods exhibit high levels of embryonic lethality relative to *mes-4* single mutants. ****P ≥ 0.0001, unpaired *t*-test. *n* ≥ 10 parental animals per genotype. Whiskers indicate min/max. (B) Representative Z projection images of H3K36me3-stained adult gonads. Proliferative zone is identified by DNA morphology (Francis *et al.* 1995) and is indicated by blue bracket in wild type. Orange arrows indicate the somatic distal tip cell, for which staining is unaffected between genotypes. Images were acquired under identical exposure times. Scale bar, 10 μm. Quantification provided in Supplementary Figure S9A. (C) MES-4::GFP dissected adult gonad stained with GFP (green) and OEF-1 (red). MES-4::GFP levels reduce in the meiotic region before becoming detectable again in late pachytene as previously reported (Fong *et al.* 2002). OEF-1 levels decrease abruptly at late pachytene (McManus and Reinke 2018). Scale bar, 10 μm.

The localization of OEF-1 to sites of H3K36me3 as well as the increased H3K27me3 levels in *oef-1* mutants suggested that OEF-1 might have a genetic interaction with the MES pathway, which establishes these two marks in germ cells. Specifically, because OEF-1 and MES-4 have opposite effects on X-linked H3K27me3, we expected that loss of OEF-1 might suppress the second-generation sterility of *mes* mutants (Capowski *et al.* 1991). To test this possibility, we created strains lacking *oef-1* as well as either *mes-4* (the HMT responsible for H3K36me3) (Bender *et al.* 2006) or *mes-2* (the catalytic subunit of PRC2 that deposits H3K27me3) (Bender *et al.* 2004). Contrary to expectation, loss of *oef-1* enhanced *mes* phenotypes. Embryonic lethality was elevated in the offspring of first-generation *oef-1; mes-4* double mutants relative to *mes-4* single mutants (Figure 5A, Supplementary Table S2) (51.6% *vs* 13.7%, *P* < 0.0001). Abnormal chromatin organization was frequently noted in the double mutant embryos, which might contribute to the lethality (Supplementary Figure S8). High levels of embryonic lethality also occurred when *oef-1* was crossed to a second *mes-4* allele (Supplementary Table S3). Consistent with the *mes-4* single mutant phenotype, *oef-1; mes-4* F2 survivors became sterile adults (Supplementary Table S4). In addition, *mes-2; oef-1* first-generation mutants showed a small but significant increase in embryonic lethality (5.55% *vs* 1.44% in *mes-2* single mutants, *P* < 0.0001) (Supplementary Table S5). *mes-2; oef-1* double mutants also exhibited an increase in the incidence of males (1.61% *vs* 0.42% in *mes-2* single mutants, *P* < 0.0001), suggesting higher rates of X chromosome nondisjunction. These results indicate that loss of OEF-1 has a greater effect in *mes-4* mutants relative to *mes-2* mutants, consistent with co-localization of OEF-1 and MES-4 at germline-expressed genes (Rechtsteiner *et al.* 2010; McManus and Reinke 2018).

We speculated that the strong genetic interaction between *oef-1 and mes-4* might be due to synergistic effects on H3K36me3 levels, and therefore performed immunostaining of H3K36me3 (Figure 5B, Supplementary Figure S9). Consistent with the ChIP-seq data, *oef-1* mutant germlines displayed H3K36me3 levels similar to wild type. *mes-4* single mutants occasionally showed a decrease in H3K36me3 staining in the proliferative zone, as reported previously (Kreher *et al.* 2018). Strikingly, *oef-1; mes-4* double mutants exhibited even stronger reduction in H3K36me3 levels, particularly in the proliferative zone, where both OEF-1 and MES-4 are present at high levels (Figure 5C, Supplementary Figure S9A). Thus, loss of OEF-1 enhanced the reduction of H3K36me3 in *mes-4* mutants specifically in the region of the germline in which the two proteins colocalize, which might underlie the enhanced phenotypes seen in the *oef-1; mes-4* mutant. We tested the specificity of this interaction by examining the relationship between OEF-1 and the only other H3K36me3 HMT in *C. elegans*, MET-1 (Kreher *et al.* 2018). *oef-1; met-1* double mutants did not display any significant alteration in H3K36me3 levels compared to either single mutant (Supplementary Figure S9B). Thus, *oef-1* specifically enhances *mes-4* but not *met-1* defects in H3K36me3 levels.

The relationship of *oef-1* with *mes-4* in maintaining H3K36me3 levels suggested that OEF-1 might function with other known H3K36me3-associated proteins. We therefore tested whether *oef-1* might also have a genetic interaction with *mrg-1*, the ortholog of another H3K36me3-associated protein, MRG15 (Iwamori *et al.* 2016). Strikingly, loss of *oef-1* also enhanced the *mrg-1* phenotype, leading to sterility one generation earlier than the *mrg-1* mutant alone (Supplementary Table S6). Thus, OEF-1 enhances the phenotypes of two factors associated with H3K36me3, reinforcing the possibility that OEF-1 acts at sites of H3K36me3 in germ cells and might influence H3K36me3-related processes.

### Loss of OEF-1 activity decreases splicing efficiency in the germline

**Figure 6:**
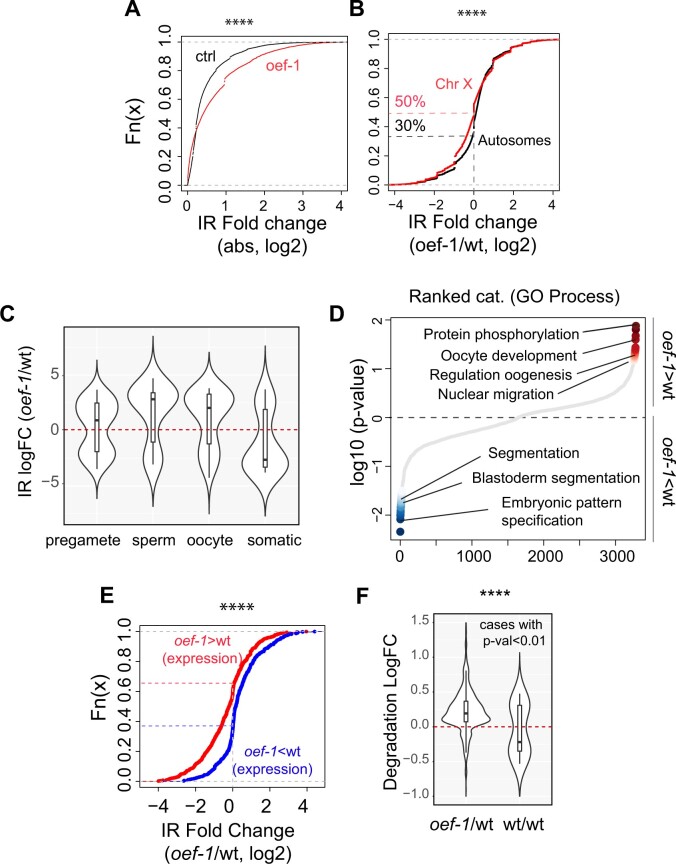
*oef-1* mutants exhibit decreased splicing integrity. (A) Cumulative plot showing an increase in intron retention in *oef-1 vs* wild type (labeled *oef-1*/wt) relative to wild type *vs* an independent wild type sample (Herbette *et al.* 2020; labeled control, ctl). Differential intron retention [IR, absolute log2 fold change (FC)] is on the *x*-axis, and accumulated incidence (cumulative frequency) on the *y*-axis for the *oef-1*/wt (red) and control (black) comparisons. *P* < 2.2e-16, Wilcox test. (B) Cumulative plot showing relative frequency of intron retention (IR) on autosomal introns compared to X-linked introns for the *oef-1*/wt dataset. The dotted lines mark log fold change = 0, showing that about 50% of X-linked genes (red) have increased intron retention in *oef-1* mutants relative to control, compared to 64% of autosomal genes (black). *P* = 0.01, Wilcoxon test. (C) Violin plots showing relative frequency of intron retention in *oef-1* mutants *vs* wild type for genes expressed in either immature germ cells (pregam) or differentiating germ cells (sperm, oocyte) or in the soma (Lee *et al.* 2017). (D) Graph of significant GO terms for genes with increased (red) or decreased (blue) intron retention in *oef-1* mutants *vs* wild type. (E) Cumulative plot showing relative frequency of intron retention for genes with either increased (red) or decreased (blue) expression in *oef-1* mutants relative to wild type (includes all genes with 0.5 log2 fold change or greater, no significance cutoff). Dotted lines show percentage of the two distributions at log FC = 0. About 66% of genes with lower expression in *oef-1* mutants also have increased intron retention, compared to only 40% of genes with higher expression in *oef-1* mutants, *P* < 2.2e-16, Wilcoxon test. (F) Violin plot showing significantly increased levels of transcript degradation in the experimental dataset relative to the control dataset, *P* = 0.03, Wilcoxon test; only cases with significant transcript degradation rate (measured with *P*-value < 0.01) are displayed.

Of the downstream molecular processes associated with H3K36me3 that might be affected by OEF-1, we focused on mRNA splicing, since OEF-1 binds across both exons and introns. Using the RNA-seq data from dissected wild type and *oef-1* mutant gonads, we analyzed the frequency of intron retention, which indicates a disruption of normal splicing events. To rigorously define the baseline variation of intron retention that might normally occur between any two independent samples, we established a control comparison between our wild-type sample and an independently collected wild-type dataset, also from dissected gonads (Herbette *et al.* 2020) (Supplementary Figure S10). We then determined the rate of intron retention between *oef-1* and our wild-type sample and found that loss of OEF-1 activity led to significantly increased intron retention compared to this control (Figure 6A). Moreover, genes with increased intron retention in *oef-1* mutants exhibit properties consistent with OEF-1 localization: they are biased toward autosomes (Figure 6B) and have preferential expression in the germline (Figure 6C). These genes encode proteins associated with germline functions (Figure 6D), including several involved in the cell cycle, meiosis, and nuclear organization (*e.g.*, *mcm-7*, *cdc-25.1*, *cya-1*, *cdc-42*, *nuc-1*, *sun-1*, *tac-1*, and *lmn-1*) that might underlie the precocious proliferation and increased apoptosis phenotypes seen upon loss of *oef-1*. Indeed, these candidate genes display OEF-1 binding (Supplementary Figure S11). By contrast, genes with more intron retention in the wild type control are biased toward genes expressed in the soma and encode proteins associated with zygotic functions. Finally, a negative correlation exists between transcript abundance and intron retention in *oef-1* mutants: genes with decreased transcript abundance are biased toward increased intron retention, whereas genes with increased transcript abundance are biased toward less intron retention (Figure 6E). This observation suggested that increased intron retention might lead to increased transcript degradation, and indeed we found that *oef-1* mutants displayed lower transcript integrity, a hallmark of RNA degradation (Figure 6F). Together these observations indicate that OEF-1 contributes to the efficient splicing of germline-expressed genes, perhaps via its association with H3K36me3.

## Discussion

Here, we describe a potential molecular role for the germline-expressed zinc-finger protein OEF-1 and provide insight into how the pathways that balance H3K36me3 and H3K27me3 in the germline can be modulated. OEF-1 associates with genes with relatively higher levels of H3K36me3 but is not essential to maintain those levels. Thus, we propose that OEF-1 might act as a “reader” of H3K36me3 or associate with such a “reader” protein (Supplementary Figure S12). Different H3K36me3 reader protein complexes can affect cryptic transcription initiation, splicing, RNA processing, chromatin remodeling, and DNA repair (Li *et al.* 2019). Indeed, we find evidence that OEF-1 does affect splicing integrity of autosomal germline-expressed genes with higher levels of H3K36me3. Notably, the genes with increased intron retention in *oef-1* mutants display a mild decrease in gene expression (Figure 6F), which could be a consequence of degradation of the aberrant transcripts (Figure 6G). Presumably, the splicing defects and changes in degradation rates would occur variably among germline-expressed transcripts, which would explain why we identified relatively few autosomal genes with statistically significant changes in gene expression (Figure 1, A and B). In addition, other RNA processing defects that we did not assay might also occur in *oef-1* mutants and contribute to the variable transcript degradation (*e.g.*, Meers *et al.* 2017).

Alteration of splicing patterns could contribute to the organismal phenotypes of precocious proliferation, faster germ cell progression, and defective synaptic checkpoint in *oef-1* mutants (McManus and Reinke 2018). Indeed, some genes with increased intron retention upon loss of OEF-1 encode proteins with cell cycle and meiotic functions. Whether OEF-1 interacts with various splicing regulators or other H3K36me3 reader proteins remains to be determined. For example, OEF-1 might associate with MRG-1, which also affects proliferation and the synaptic checkpoint in the germline (Takasaki *et al.* 2007; Dombecki *et al.* 2011), and indeed we found that loss of OEF-1 enhances *mrg-1* mutant sterility. In addition, even though OEF-1 associates with chromatin, it might do so indirectly through an interaction with nascent mRNA undergoing splicing. OEF-1 has a single C2H2 zinc finger domain, which might interact with either RNA or DNA, and an unstructured, possibly intrinsically disordered domain of about 80 amino acids at the C terminus, which might help in forming protein complexes. Systematic identification of OEF-1-interacting proteins will be essential for understanding how it reads or transmits the information from H3K36me3 patterns in the germline to affect downstream processes.

Based on our current genetic and molecular data, we suggest that OEF-1 might stabilize H3K36me3 levels, perhaps by limiting access by demethylases (Supplementary Figure S12). Decreased H3K36me3 is not detected in *oef-1* mutants as long as MES-4, the HMT that actively maintains pre-existing H3K36me3 patterns (Rechtsteiner *et al.* 2010), is present. However, the absence of both MES-4 and OEF-1 leads to a substantial decrease in H3K36me3 levels compared to the loss of either alone. In this model, we speculate that the absence of OEF-1 might cause elevated MES-4 activity to maintain normal levels of H3K36me3 at existing sites. MES-4 repels PRC2 activity, concentrating it to the X and other silenced genes (Gaydos *et al.* 2012). Thus, in *oef-1* mutants, increased MES-4 activity at its normal sites of action could indirectly result in increased H3K27me3 on the X and at “silenced” autosomal genes. The milder phenotype noted in *mes-2; oef-1* mutants relative to *oef-1; mes-4* mutants is consistent with this idea. *mes-2* mutants already lack detectable H3K27me3 in germ nuclei (Bender *et al.* 2004), and as such the loci that typically have this modification would be minimally affected in *mes-2; oef-1* mutants. In this way, OEF-1 could influence the levels of histone modifying activity at both active and “silenced” genes in the germline. Future genomic studies will determine at high resolution how these competing histone marks are altered when the activity of both OEF-1 and the associated HMT complexes are simultaneously disrupted.

## Data availability

The gene expression omnibus (GEO) accession number for the ChIP-seq and RNA-seq datasets reported in this paper is GSE147401. Previously published OEF-1::GFP ChIP-seq is available under accession number GSE107190 (McManus and Reinke 2018). Supplementary material is available at figshare: https://doi.org/10.25387/g3.16586714.
